# Notes and News

**Published:** 1895-08-10

**Authors:** 


### Notes and News.

(Collected and Communicated.)

Ten medical men are numbered amongst the members of the new Parliament.

The Queen has sent a donation of £100 to the building fund of the Royal Free Hospital, Gray's Inn Road, the new front buildings of which were recently opened by the Prince and Princess of Wales.

Owing to the Duke of Devonshire having received a summons from Her Majesty to go to Osborne on Wednesday the ceremony of opening the new Home for Epileptics at Chalfont St. Peter has been postponed.

The Pasteur filter, which is stated to have been most successfully used for some time past in the French army, is now to be introduced at Darjeeling. The Commissioners are about to cause the filters to be used in connection with their water mains.

The Governors of the Midland Counties Sanatorium have accepted Colonel Wilkinson's gift of the Royal Hotel and grounds at Sutton Coldfield for conversion into a sanatorium or convalescent home. It is to be for the benefit of clerks, shop assistants, and persons occupied in similar occupations.

Surgeon-Captain Whitchurch, the new V.C., received his decoration from the Queen's own hands last week. The command to attend at Osborne was sent at so short a notice that Dr. Whitchurch was unable to appear in uniform, Her Majesty decreeing that he might attend in plain apparel.

T seems almost incredible that the founder of the Geneva Convention, the origin of the Red Cross societies scattered now over so many countries, is in actual want of daily necessities. Yet such is stated to be the case in respect to Henry Dunant, now sixty-seven years of age. He is said to have given up all his property in the cause of humanity, and it would be a reproach indeed to the humanity to which he has devoted his possessions and his energies if it allowed him to remain in want one day after his sad circumstances become known.

Appeal is made on behalf of the Seaside Camp for London Working Boys. Last year 11,530 boys from fourteen to seventeen years of age were under canvas at Romney, in Kent. They pay five shillings themselves per week, but five shillings only covers the cost of railway fare, and consequently the managers have to look to the public for support and sympathy in their work.

Sir Joseph Lister received his portrait from his past colleagues and pupils on the 31st ult. The chairman on the occasion, Dr. W. Playfair, said that the presentation was to be regarded as a mark of personal affection and esteem on the part of the donors, and not as a public recognition of services which deserved international honours. Sir Eric Erichsen presented the portrait, painted by Mr. J. Lorrimer, to Sir Joseph Lister, who, in accepting the gift, briefly sketched the steps by which he had arrived at his conclusions as to the theory of antiseptic surgery.

An International Medico-Legal Congress will be held in New York on September 4th, 5th, and 6th, under the chairmanship of Ex-Surrogate Rastus Ransom. The congress will be divided for the consideration of the following subjects: Psychology and Psychologic Medicine, Inebriety, Sociology and Criminology, Experimental Psychology and Medical Jurisprudence, &c.

We now learn that the applications for admission to the New School of Medicine for Women at St. Petersburg are very numerous; so numerous indeed are they that the Russian Education Department have decided on the admission of female medical students to the medical faculties of all universities in the empire. The New School of Medicine at St. Petersburg is to be opened at the beginning of the academic year 1895-6.

A few more cases of plague are reported from Hong Kong, but owing to the prompt measures taken it is to be hoped no further outbreak will follow. The patients were at once removed to the Kennedy Town Hospital, which has been kept ready for any such emergency, and their houses submitted to a thorough cleansing and disinfecting, the other members of the contaminated families being sent in quarantine on boats for ten days.

Increased fever accommodation is much needed at Huddersfield. At a recent meeting of the Town Council it was pointed out that all the wards at present available were now required for scarlet fever cases, and it had, therefore, been impossible for some time to admit cases of typhoid and diphtheria, which was a matter for serious consideration. The medical officer also drew attention to the fact that an increase in population would surely follow an improvement in the staple industries, and an institution of fifty-eight beds might be shortly called upon to minister to the wants of a population of over 100,000. It is to be hoped that the committee will lose no time in carrying out their intention of erecting the extra accommodation necessary on the site secured for hospital purposes. The importance of adequate provision for the treatment and isolation of infectious diseases cannot be overrated, and every moment lost in securing it is to be deplored.

